# Clinical implications of elevated serum interleukin-6 in IgG4-related disease

**DOI:** 10.1371/journal.pone.0227479

**Published:** 2020-01-17

**Authors:** Satoshi Tsukuda, Tsukasa Ikeura, Takashi Ito, Koh Nakamaru, Masataka Masuda, Yuichi Hori, Manami Ikemune, Masato Yanagawa, Toshihiro Tanaka, Takashi Tomiyama, Takashi Yamaguchi, Yugo Ando, Kazushige Uchida, Toshiro Fukui, Akiyoshi Nishio, Rika Terasawa, Noboru Tanigawa, Kazuichi Okazaki

**Affiliations:** 1 Division of Gastroenterology and Hepatology, The Third Department of Internal Medicine, Kansai Medical University, Hirakata, Osaka, Japan; 2 Department of Gastroenterology and Hepatology, Kochi Medical School, Kochi University, Nankoku, Kochi, Japan; 3 Department of Radiology, Kansai Medical University, Hirakata, Osaka, Japan; National Institute of Dental and Craniofacial Research, UNITED STATES

## Abstract

**Introduction:**

Some patients with IgG4-related disease (IgG4-RD) exhibit elevated serum interleukin (IL)-6 with excessive inflammatory reactions or with repeating relapse. To date few reports pertaining to clinical implications of elevated serum IL-6 in IgG4-RD patients have been published. The aims of the current retrospective study were to investigate the clinical implications of elevated serum IL-6 in IgG4-RD patients, and to examine whether IL-6 can predict the activity and/or relapse of the disease.

**Materials and methods:**

We examined the clinical picture at the onset of 43 patients who were diagnosed with IgG4-RD in our hospital and were able to measure serum IL-6 before steroid treatment.

**Results:**

The median level of serum IL-6 was 2.2 pg/mL. There was a significant correlation between IL-6 and C-reactive protein (CRP) level (r = 0.397, *p* = 0.008), hemoglobin level (r = -0.390, *p* = 0.010) and albumin level (r = -0.556, *p* < 0.001). When 43 patients were divided into two groups by using a cut-off IL-6 of 4 pg/mL, the high IL-6 group showed higher age, lower albumin, higher CRP and higher aspartate aminotransferase (AST) (age *p* = 0.014, albumin *p* = 0.006, CRP *p* <0.001, AST *p* = 0.009). Hepatic swelling and splenomegaly were significantly more prevalent in the high IL-6 group than it was in the low IL-6 group (liver *p* < 0.001, spleen *p* = 0.020). Biliary tract involvement tended to admit more in the high IL-6 group (*p* = 0.060).

**Conclusion:**

Serum IL-6 level at the onset of IgG4-RD may be significantly correlated with clinical inflammatory parameters and it may also be associated with involvement of the bile duct, liver, and spleen.

## Introduction

IgG4-related disease (IgG4-RD) is a systemic condition that is histologically characterized by abundant infiltration of IgG4-positive cells and lymphocytes in conjunction with massive fibrosis, particularly storiform fibrosis. It evidently involves a wide range of organs including the pancreas, bile duct, lacrimal glands, salivary glands, central nervous system, thyroid, lungs, liver, gastrointestinal tract, kidneys, prostate, retroperitoneum, aorta/artery, lymph nodes, skin, and breasts [1]-[2]. The radiological characteristics of IgG4-RD include diffuse or focal organ enlargement and mass-forming or nodular/thickened lesions in the affected organs, and patients with IgG4-RD frequently exhibit elevated serum IgG4.

The prognosis of IgG4-RD is considered favorable if substantial improvements in serological and radiological abnormalities are observed after steroid therapy; however, approximately 31%–57% of patients experience relapse of the disease during steroid tapering or after steroid cessation [3]-[4]. To ensure a correct diagnosis of IgG4-RD, the patient’s condition should be differentiated from other disorders including sarcoidosis, Castleman’s disease, Wegener’s granulomatosis, lymphoma, and cancer [2]. Patients with plasma cell-type or mixed-type multicentric Castleman’s disease (MCD) exhibit abundant IgG4-positive plasmacyte infiltration with high serum IgG4, which are also commonly observed in IgG4-RD patients. Notably however they also exhibit quite different systemic manifestations such as fever, fatigue, and loss of appetite and weight, and abnormal laboratory findings including elevated C-reactive protein (CRP), anemia, hypoalbuminemia, hypocholesterolemia, and thrombocytosis with poor prognosis, which are reportedly associated with a condition known as hyper- interleukin (IL)-6 syndrome that is characterized by elevated serum IL-6. IL-6, a principal mediator in inflammatory conditions, is reportedly a marker that is indicative of severity, progression, and outcome in several diseases [5]-[6]. Although MCD and IgG4-RD are thought to be distinct clinical entities, some patients with IgG4-RD exhibit elevated serum IL-6 [7]-[8], and in such patients it can be difficult to distinguish their condition from MCD.

To date few reports pertaining to clinical implications of elevated serum IL-6 in IgG4-RD patients have been published. The aims of the current retrospective study were to investigate the clinical implications of elevated serum IL-6 in IgG4-RD patients, and to examine whether IL-6 can predict the activity and/or relapse of the disease.

## Materials and methods

The study was approved by the Ethics Committee of Kansai Medical University. Written informed consent from patients was obtained. Of all 129 IgG4-RD patients included in our database since 2002, we enrolled 38 patients diagnosed as definitive or probable cases of type 1 AIP according to the International Consensus Diagnostic Criteria for AIP and 5 patients diagnosed as cases of definitive, probable, or possible IgG4-RD according to the comprehensive diagnostic criteria for IgG4-RD [2] in the current study. Consequently, we analyzed data of a total of 43 patients.

Gender and age data were retrospectively collected for each patient, as were laboratory and radiological data at clinical onset of IgG4-RD prior to treatment including white blood cell, neutrophil, eosinophil, and lymphocyte counts, hemoglobin, IL-6, total IgG, IgG4, IgE, CRP, amylase, blood glucose, total protein, albumin, aspartate aminotransferase (AST), alanine aminotransferase (ALT), alkaline phosphatase (ALP), total bilirubin and creatinine levels, medical history, and details of prior therapy for the disease (steroid and/or immunosuppressant drugs, no treatment) and/or disease relapse.

Serum samples for measuring IL-6 levels were obtained after centrifugation (2000 rpm, 10 minutes, 4°C) and stored at -80°C. IL-6 levels were measured by chemiluminescent enzyme immunoassay (Human IL-6 CLEIA Fujirebio; Fujirebio, Inc., Tokyo, Japan). The reference value of IL-6 was 4.0 pg/mL or less based on the manufacturer’s instruction. The reference values of the other items of blood test were as follows: white blood cell, 8500–3500 /μL; Eosinophil, 35–450 /μL; hemoglobin, 11.3–15.4 g/dL; albumin, 3.8–5 g/dL; CRP, <0.3 mg/dL; AST, 13–35 U/L; ALT, 5–35 U/L; ALP, 107–340 U/L; total bilirubin, 0.2–1.2 mg/dL; IgG, 870–1700 mg/dL; IgG4, 4.8–105 mg/dL; C3, 69–128 mg/dL; C4, 14–36 mg/dL; CH50 30–50 U/mL; and IgE, < 320 IU/mL.

Three experts in IgG4-RD and AIP including a radiologist independently assessed the presence or absence of morphological alterations of the pancreas, intrahepatic and extrahepatic bile duct, lacrimal glands, salivary glands, thyroid, lungs, liver, spleen, kidneys, retroperitoneum, arteries, and lymph nodes based on computed tomography, magnetic resonance imaging, and endoscopic retrograde cholangiopancreatography with and without intraductal ultrasound at the time of clinical onset. When an organ exhibited enlargement, mass-formation, or thickened lesions, it was classified as affected.

Diabetes mellitus was diagnosed in accordance with the criteria established by the committee of the Japanese Diabetes Society [9]. Initial steroid therapy was performed with either conventional oral steroid therapy or steroid pulse therapy [10]-[11]. Conventional oral steroid therapy consisted of prednisolone administrated at an initial dose of 30–40 mg per day for 2 weeks, which was subsequently gradually tapered by 5 mg over several weeks until the maintenance dosage was reached. Steroid pulse therapy consisted of intravenous administration of methylprednisolone at a dosage of 125–500 mg per day for 3 consecutive days per week for 2 consecutive weeks. After steroid pulse therapy prednisolone was administered at 20 mg per day, and it was gradually tapered until the maintenance dosage was reached. After initial steroid therapy, if needed, maintenance steroid therapy was administered for at least 6 months with prednisolone at a dosage of 2.5–5.0 mg per day to prevent relapse. In some patients the immunosuppressant drug azathioprine was administered to prevent relapse. Relapse of IgG4-RD and AIP was defined as the radiological reappearance of organ involvement without suspicion of cancer during steroid treatment or after steroid withdrawal.

Continuous values were presented as the median (interquartile range). Statistical differences pertaining to continuous variables were assessed using the appropriate non-parametric test depending on the normality or otherwise of the data distribution. Correlations between the parameters were assessed using Spearman’s correlational analysis. Mann-Whitney test was used in comparisons between two groups. *χ*^2^ test was used for the comparison of proportions. Values of *p* < 0.05 (two-sided) were considered statistically significant. Statistical analyses were performed using JMP 13.2.0 software (SAS Institute, Inc., Cary, NC).

## Results

### Patient characteristics

Characteristics of the 43 patients with IgG4-RD in the current study are shown in Tables 1 and 2. The median levels of serum IL-6, CRP, and IgG4 were 2.2 pg/mL, 0.10 mg/dL, and 290 mg/dL, respectively (Table 1). The affected organs associated with IgG4-RD were the lacrimal gland (9%, *n* = 4), salivary gland (37%, *n* = 16), pancreas (86%, *n* = 37), biliary tract (63%, *n* = 27), liver (9%, *n* = 4), spleen (16%, *n* = 7), kidney (19%, *n* = 8), retroperitoneum (9%, *n* = 4), lymph node (49%, *n* = 21), and aorta (2%, *n* = 1) (Table 2). Of 43 patients who underwent steroid therapy as initial treatment, 15 (36%) experienced relapse of the disease (Table 1). Characteristics of 10 patients with elevated serum IL-6 are shown in Table 3.

**Table 1:** The clinical features of IgG4-RD patients included in the current study.

	All patients (n = 43)
Age, y	66 (55–71)
Gender, male, n (%)	33 (77%)
Diabetes, n (%)	11 (26%)
Laboratory examination	
IL-6, pg/mL	2.2 (1.3–3.8)
IL-6 increase (> 4 pg/mL), n (%)	10 (23%)
CRP, mg/dL	0.10 (0.05–0.30)
CRP increase (> 0.3 mg/dL), n (%)	10 (23%)
IgG, mg/dL	1723 (1362–2185)
IgG increase (> 1700 mg/dL), n (%)	21 (41.8%)
IgG4, mg/dL	290 (204–578)
IgG4 increase (> 135 mg/dL), n (%)	37 (86%)
IgE, IU/mL	515 (311–1254)
IgE increase (> 320 IU/mL), n (%)	20 (46.5%)
WBC, /μl	5300 (4500–6600)
WBC increase (> 8500 /μL), n(%)	2 (5%)
Eosinophil, /μl	213 (110–281)
Eosinophil increase (> 5% of WBC count), n (%)	7 (16.3%)
Relapse, n (%)	15/42 (36%)

IL-6: Interleukin-6, CRP: C-reactive protein, WBC: White blood cells

Continuous data values are presented as the median (interquartile range).

**Table 2:** Prevalence of involvement of each organ and serum levels of IL-6 and IgG4.

Organ involvement		IL-6, pg/mL	IgG4, mg/dL
Lacrimal gland, n (%)	4 (9%)	1.4 (0.9–2.4)	614 (193–1052)
Salivary gland, n (%)	16 (37%)	1.5 (1.1–2.2)	478 (290–811)
Pancreas, n (%)	37 (86%)	2.3 (1.3–4.7)	285 (202–478)
Biliary tract, n (%)	27 (63%)	2.7 (1.8–5.9)	243 (188–480)
Liver, n (%)	4 (9%)	20.6 (5.5–76.8)	642 (184–1133)
Spleen, n (%)	7 (16%)	4.9 (2.0–34.0)	498 (204–1080)
Kidney, n (%)	8 (19%)	3.8 (2.2–7.7)	522 (251–809)
Retroperitoneum, n (%)	4 (9%)	2.3 (1.7–2.9)	491 (304–717)
Lymph node, n (%)	21 (49%)	1.8 (1.1–3.6)	485 (265–943)
Aorta/artery, n (%)	1 (2%)	3.0	244

IL-6: Interleukin-6

Continuous data values are presented as the median (interquartile range).

**Table 3:** Clinical features of 10 patients with elevated IL-6.

Case	Gender	Age (y)	IL-6 (pg/mL)	WBC (/μL)	Hb (g/dL)	Alb (g/dL)	CRP (mg/dL)	IgG4 (mg/dL)	Diagnosis according to ICDC or CCD	relapse	Organ involvement
1	Female	71	91.1	4000	8.8	2.5	3.75	177	Definite	-	Pancreas, Biliary tract, Kidney, Lymph node, Liver, Spleen
2	Female	64	34	10400	7.8	1.5	5.05	1080	Definite	+	Pancreas, Lymph node, Liver, Spleen
3	Male	60	12.3	4700	11.5	N/A	2.27	83.8	Possible	-	Pancreas, Biliary tract
4	Male	69	7.9	5600	12.4	3.7	0.38	465	Definite	-	Pancreas, Biliary tract, Salivary gland, Kidney
5	Male	73	7.2	8100	11.8	3.2	0.60	204	Definite	-	Pancreas, Biliary tract, Kidney, Liver, Spleen
6	Male	53	6.9	7100	12.9	3.9	0.18	244	Definite	-	Pancreas, Biliary tract, Lymph node
7	Male	81	6.6	6400	14.3	3.6	0.11	133	Definite	+	Pancreas, Biliary tract
8	Male	74	6.2	3700	11.7	3.5	0.30	120	Possible	-	Pancreas, Biliary tract
9	Male	84	4.9	7500	13.7	N/A	0.60	1150	Definite	+	Pancreas, Biliary tract, Kidney, Lymph node, Liver, Spleen
10	Male	73	4.4	5300	14.2	3.2	0.82	234	Definite	-	Pancreas, Biliary tract

IL-6: Interleukin-6, WBC: White blood cells, Hb: Hemoglobin, Alb: Albumin, CRP: C-reactive protein

N/A: Not available

**Fig 1:**
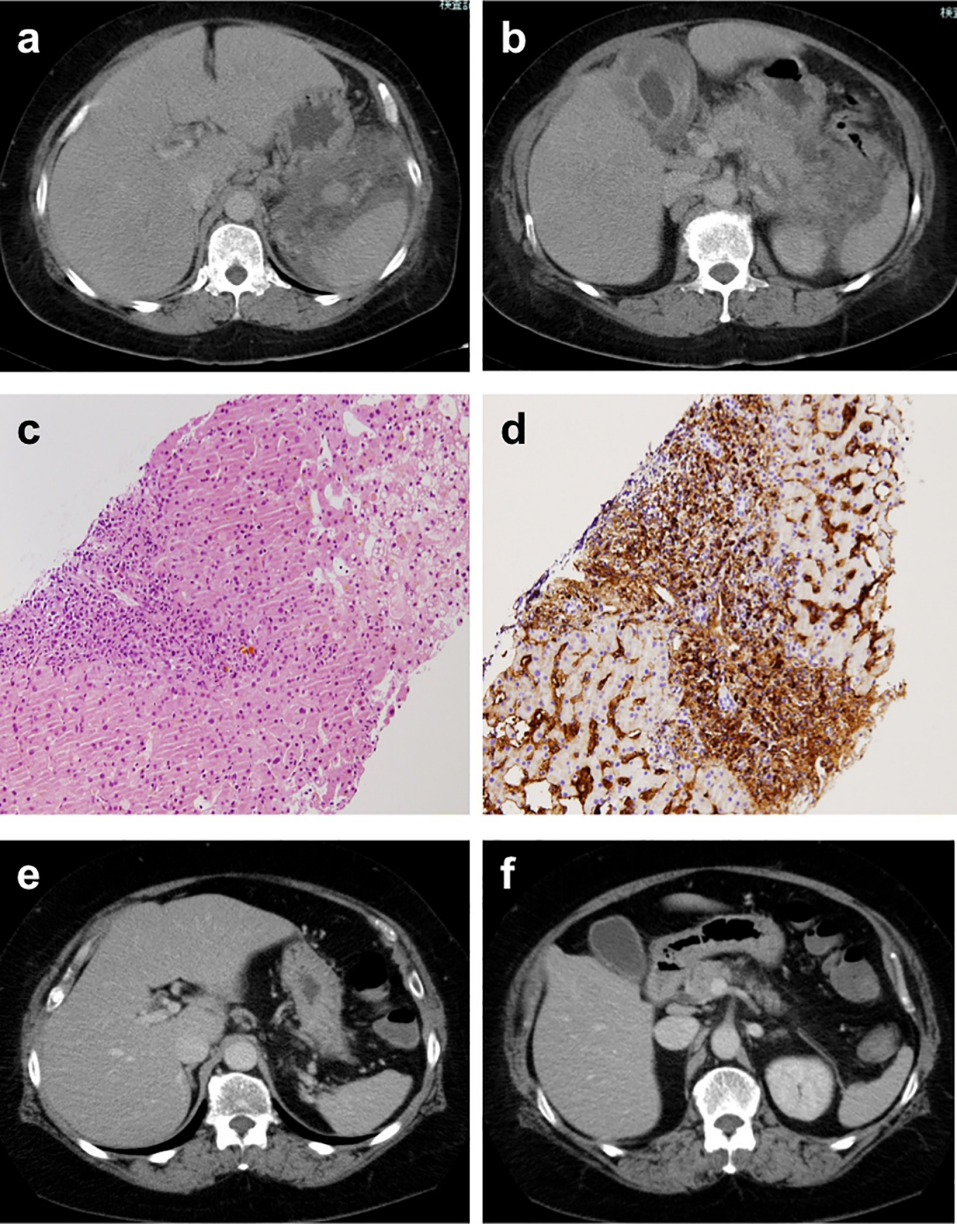
CT images of our patient diagnosed as IgG4-RD who has with elevated serum IL-6 levels. a, b: CT at the onset of the disease shows marked wall thickening of the gallbladder, hepatomegaly, fluid retention around the pancreas, and paraaortic and splenic hilar lymphadenopathy are seen. c: A histological examination of a biopsy sample from the liver showing abundant infiltration of lymphocytes in the portal tract (Hematoxylin and Eosin staining). Follicular lymphoid hyperplasia is not evident. d: Immunohistochemically dense infiltration of IgG4-positive plasma cells is evident. e, f: CT after steroid therapy shows the resolution of abnormal manifestations.

Fig 1 shows CT images from a 63-year-old woman who complained of recurrent fever and exhibited elevated serum IL-6 (34 pg/mL) and was diagnosed with IgG4-RD [8]. Laboratory data at onset revealed anemia (hemoglobin, 7.8 g/dL), hypoalbuminemia (albumin, 1.5 g/dL), and elevated serum levels of IgG4 (1080 mg/dL) and CRP (5.05 mg/dL). Imaging revealed marked wall thickening of the gallbladder, hepatomegaly, mild pancreatic swelling with peri-pancreatic effusion, and splenic hilar lymph nodes (Fig 1A and 1B). Histological examination of liver biopsy specimens revealed infiltration of numerous neutrophil cells was evident in the HE staining and dense infiltration of IgG4-positive plasma cells (IgG/IgG4 ratio > 40%) with fibrosis in the portal area (Fig 1C and 1D). She was ultimately diagnosed with IgG4-RD in accordance with comprehensive diagnostic criteria [2], though some common clinical manifestations of MCD were present. After the initiation of steroid therapy, her symptoms including fever and the abnormalities evident via imaging resolved (Fig 1E and 1F). Eventually, her laboratory data in remission were as follows; IL-6, 3.0 pg/mL; IgG4, 186 mg/dL; hemoglobin, 11.2 g/dL; ALB, 4.2 g/dL; and CRP, 0.23 mg/dL.

### Serum IL-6 and hematological parameters

**Fig 2:**
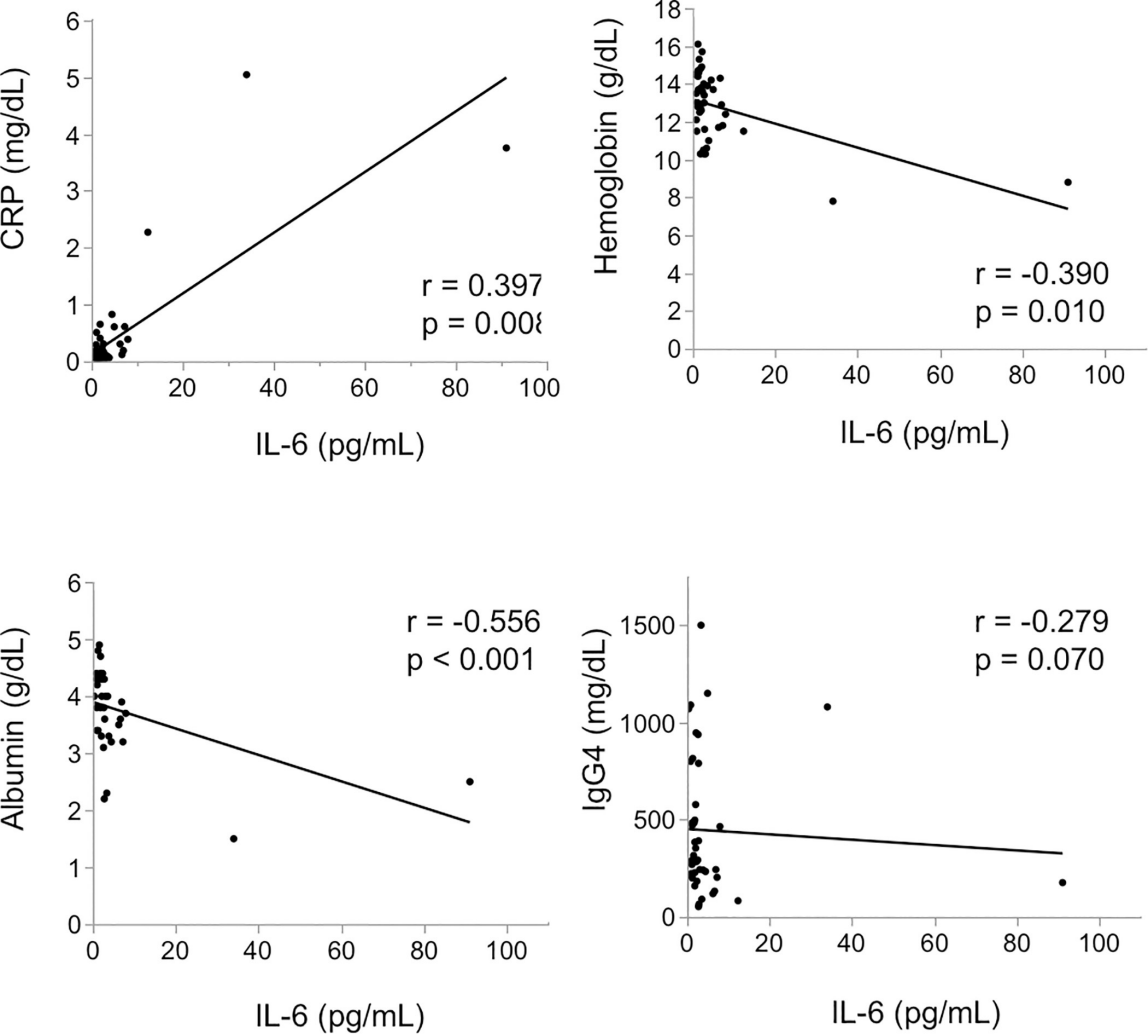
The correlative relationship between the serum IL-6 levels and CRP, hemoglobin, albumin, and IgG4. Bold lines are fitted curve. There was a positive significant correlation between IL-6 and CRP (a) and a negative significant correlation between Hemoglobin (b) and Albumin (c). There was no significant correlation with IgG4 (d).

Fig 2 shows correlations between serum IL-6 and parameters reflecting disease activity including CRP, hemoglobin, albumin, and serum IgG4. There was a significant positive correlation between IL-6 and CRP level (r = 0.397, *p* = 0.008). IL-6 level was significantly correlated with hemoglobin level (r = -0.390, *p* = 0.010) and albumin level (r = -0.556, *p* < 0.001). There was no significant correlation between IL-6 level and IgG4 level (r = -0.279, *p* = 0.070).

### Clinical effects of serum IL-6 at the time of IgG4-RD onset

To investigate correlations between serum IL-6 and clinical manifestations at the time of IgG4-RD onset, 43 patients with IgG4-RD were divided into two groups using a reference value of IL-6: a ≤ 4.0 pg/mL group (*n* = 33), and a > 4.0 pg/mL group (*n* = 10) (Table 4). The high IL-6 group exhibited higher age, lower albumin, and higher CRP and AST levels than the low IL-6 group, and there were statistically significant differences in these parameters between the two groups (age *p* = 0.014, albumin *p* = 0.006, CRP *p* < 0.001, AST *p* = 0.009).

**Table 4:** Comparison of the clinicopathological features in patients with IgG4-RD between low and high IL-6 groups.

	Low IL-6 group (n = 33)	High IL-6 group (n = 10)	*p* value
Age, y	63 (51–68)	72 (63–76.5)	0.014
Gender, male	25 (75.8%)	8 (80%)	0.781
Diabetes mellitus	7 (21.2%)	4 (40%)	0.233
Laboratory data			
WBC, /μL	5200 (4500–6300)	6000 (4525–7650)	0.307
Hb, g/dL	13.0 (11.9–14.5)	12.1 (10.8–13.8)	0.139
Alb, g/dL	4.0 (3.5–4.4)	3.4 (2.7–3.7)	0.006
CRP, mg/dL	0.06 (0.05–0.15)	0.60 (0.27–2.64)	< 0.001
AST, U/L	23 (19–35)	46 (26–98)	0.009
ALT, U/L	22 (16–43)	37 (18–208)	0.227
ALP, U/L	292 (194–455)	490 (264–785)	0.092
T-Bil, mg/dL	0.6 (0.6–1.3)	1.6 (0.6–4.2)	0.056
IgG, mg/dL	1720 (1407–2068)	2073 (1180–3839)	0.571
IgG4, mg/dL	316 (226–684)	219 (130–619)	0.201
C3, mg/dL	85 (76–133)	68 (47–143)	0.389
C4, mg/dL	19 (12–25)	12 (2–25)	0.186
CH50, U/mL	54 (48–59)	81 (10–86)	0.332
Organ involvement			
Pancreas	27 (82%)	10 (100%)	0.146
Biliary tract	19 (58%)	9 (90%)	0.060
Lacrimal gland	4 (12%)	0 (0%)	0.248
Salivary gland	15 (46%)	1 (10%)	0.042
Lymph node	17 (52%)	4 (40%)	0.523
Retroperitoneum	4 (12%)	0 (0%)	0.248
Liver	0 (0%)	4 (40%)	< 0.001
Spleen	3 (9%)	4 (40%)	0.020
Kidney	4 (12%)	4 (40%)	0.047
Aorta/artery	1 (3%)	0 (0%)	0.578
Number of organ involvement			
≥ 2	29 (88%)	10 (100%)	0.248
≥ 3	19 (58%)	6 (60%)	0.892
≥ 4	9 (27%)	4 (40%)	0.443

Continuous data values are presented as the median (interquartile range).

IL-6: Interleukin-6, WBC: White blood cells, Hb: Hemoglobin, Alb: Albumin, CRP: C-reactive protein, AST: Aspartate aminotransferase, ALT: Alanine aminotransferase, ALP: Alkaline phosphatase, T- Bil: Total bilirubin

Salivary gland involvement was more frequent in the low IL-6 group (46%) than it was in the high IL-6 group (10%) (*p* = 0.042). In contrast, involvement of kidney, liver, and spleen was more frequently seen in the high IL-6 group than it was in the low IL-6 group (kidney 40% vs. 12%, *p* = 0.047, liver 40% vs. 0%, *p* < 0.001, spleen 40% vs. 9%, p = 0.020). A higher proportion of patients having biliary tract involvement in the high IL-6 group (90%) was higher than that in the low IL-6 group (58%), but the differences were not statistically significant (*p* = 0.060).

### Serum IL-6 as a predictor of relapse

To evaluate the predictive capacity of serum IL-6 with regard to relapse, We compared IL-6 levels at the clinical onset of the disease between relapse and non-relapse group after being separately divided into the population of patients who received low-dose maintenance steroid therapy (patients in whom a low dose prednisolone was continued after gradual taper of initial steroid therapy) and patients who did not received it (patients in whom prednisolone was withdrawn after gradual taper of initial steroid therapy) because a recent study reported the relapse rate varied between patients who underwent low-dose maintenance steroid therapy and patients who did not undergo the therapy [12]. In patients who received low-dose maintenance steroid therapy (Table 5) as well as patients who did not receive it (Table 6), there were no statistically significant differences in the median observation periods and IL-6 levels between relapse and non-relapse group.

**Table 5:** Comparison of IL-6 levels at the onset in patients who received maintenance therapy between relapse and non-relapse group.

	Relapse (n = 10)	Non-relapse (n = 13)	*p* value
Observation period, mo	22.5 (14–49)	44 (20–69)	0.925
IgG4, mg/dL	869 (354–1090)	228 (177–244)	< 0.001
IL-6, pg/mL	2.3 (1.1–3.3)	3.8 (1.8–6.9)	0.292

IL-6: Interleukin-6

Continuous data values are presented as the median (interquartile range).

**Table 6:** Comparison of IL-6 levels at the onset in patients who did not receive maintenance therapy between relapse and non-relapse group.

	Relapse (n = 4)	Non-relapse (n = 10)	*p* value
Observation period, mo	50 (17–80.5)	25.5 (9–29)	0.322
IgG4, mg/dL	353.5 (178–491)	379 (200–485)	0.888
IL-6, pg/mL	1.7 (1.3–4.2)	2.2 (1.2–2.5)	0.478

IL-6: Interleukin-6

Continuous data values are presented as the median (interquartile range).

## Discussion

IL-6 plays central roles in immune responses. In inflammation, IL-6 promotes the expansion and activation of T cells and the differentiation of B cells, and modulates the synthesis of positive acute phase reactants such as CRP and fibrinogen, and negative acute phase reactants such as albumin. There is also growing evidence that serum IL-6 levels can be disease severity markers and/or prognostic predictors, not only in acute inflammatory conditions such as acute pancreatitis and infection-related disorders such as sepsis but also in chronic diseases such as rheumatoid arthritis and chronic heart failure [5]-[13]. In the current study serum IL-6 level was strongly correlated with various clinical inflammatory parameters (serum CRP, hemoglobin, and albumin levels), and the prevalence of anemia, hypoalbuminemia, and CRP elevation were significantly greater in the higher IL-6 group than in the other two groups. The results suggest that serum IL-6 levels reflect clinical manifestations related to acute inflammatory reaction at the time of IgG4-RD onset. We are convinced that it is meaningful to investigate the relationship between IL-6 and the degree of acute inflammatory reaction in patients with IgG4-RD because there are very few published data about clinical implications of serum IL-6 levels in IgG4-RD patients.

Previous studies suggest that baseline elevations in serum IgG4 are predictive of IgG4-RD relapse [14]-[15]. We hypothesized that serum IL-6 levels would be a more reliable predictor of relapse after treatment than serum IgG4 levels; however, there were no significant differences in serum IL-6 levels at the onset between relapse and non-relapse group.

MCD is a polyclonal lymphoproliferative disorder caused by hyperproduction of IL-6 in affected lymphoid tissues. Patients commonly exhibit fever, night sweats, weight loss, lymphadenopathy, ascites, pleural effusion, and hepatosplenomegaly, primarily via the action of IL-6. Serologically, increased CRP and polyclonal increases in immunoglobulins are usually observed. Although the disease typically takes an aggressive course, the humanized IL-6 receptor antibody tocilizumab and a chimeric monoclonal antibody that binds to IL-6, siltuximab, are effective in MCD patients [16]-[17].

Some investigators have reported difficulty differentiating between MCD and IgG4-RD due to similar clinical and histopathological observations [18]-[19]. Patients with MCD occasionally exhibit elevated serum IgG4 and marked infiltration of IgG4-positive cells in the lymph nodes and organs involved, which would contribute to a diagnosis of IgG4-RD according to the aforementioned comprehensive diagnostic criteria for IgG4-RD [2]. Some patients diagnosed with IgG4-RD exhibit severe clinical manifestations such as fever and excessive exacerbation of laboratory parameters associated with systemic inflammation such as anemia, hypoalbuminemia, and elevated CRP. Although IL-6 immunostaining and serum IL-6 and CRP levels are reportedly useful for distinguishing between IgG4-RD and MCD [20]-[21], some researchers have suggested that IgG4-RD and MCD constitute potentially overlapping disease entities, and have alluded to an “MCD-like” subtype of IgG4-RD [7, 22]. We are convinced that all patients enrolled in the current study belonged to the disease spectrum of IgG4-RD, because all of them achieved clinical remission after steroid therapy without anti-IL-6 blockade.

In addition to MCD, anti-neutrophil cytoplasmic antibody (ANCA)-associated vasculitis (AAV) is also a disease that sometimes has difficulty in differential diagnosis of IgG4-RD because of similarity of clinical and pathological features. AAV is characterized by pauci-immune necrotizing vasculitis involving systemic or focal small vessels. In general, patients with AAV have fever, increase of inflammatory markers such as CRP and erythrocyte sedimentation rate, and symptoms resulting from the affected organ(s). AAV includes microscopic polyangiitis, granulomatosis polyangiitis (GPA; viz, Wegener’s granulomatosis), and eosinophilic GPA (EGPA; vis, Churg-strauss syndrome). Kawashima et al. reported some patients with AAV have similar features of IgG4-RD in elevation of serum IgG4 levels, distribution of organ involvement, response to steroid, and infiltration of IgG4-positive cells in the affected tissue [23]. Indeed, the comprehensive diagnostic criteria for IgG4-RD state differentiating these diseases from IgG4-RD is needed for correct diagnosis [2]. Furthermore, another recent study by Kasashima et al. revealed IgG4-RD patients with vascular lesions more often showed elevated serum IgG4 levels and low-grade fever compared to IgG4-RD patients without vascular lesions [24]. In IgG4-RD patients with vascular lesions, moreover, numerous immunopositive cells for IL-6 were observed in tissue samples obtained from the affected organ. From the results, they speculated IL-6 synthesis in IgG4-RD with vascular lesions would be related to its pathogenesis and/or progression. In our study, there was only a single patient with thickening of the aorta whose serum levels of IL-6 were not elevated; therefore, it was impossible to evaluate the validity of their results.

In a previous systematic literature review the majority of patients with MCD exhibited hepatomegaly and/or splenomegaly (78%) [25], whereas swelling of these organs is evidently rarely observed in IgG4-RD patients [26]. Isolated liver involvement with IgG4-positive plasma cell infiltration has recently been reported in patients with autoimmune hepatitis (AIH), in a condition referred to as “IgG4-associated AIH” [27]. Of all patients suffering from AIH, collective reports suggest that 3–35% have IgG4-associated AIH [27]-[28]. Because hepatic inflammation—especially that with an acute presentation—is responsible for hepatomegaly [29], patients with IgG4-associated AIH may exhibit accompanying swelling of the liver, but the associated details are not yet clear due to a lack of published data. In the current study IgG4-RD patients with high IL-6 levels were more likely to exhibit liver involvement as suggested by elevated AST levels and hepatic swelling. Considering the tendency of higher ALP levels and higher prevalence of biliary tract involvement in the high IL-6 group, elevated serum IL-6 in IgG4-RD patients may originate from the hepatobiliary system, because IL-6 is significantly up-regulated by biliary epithelial cells, hepatocytes, and liver macrophages (Kupffer cells) in response to a variety of pathcomological conditions [30]-[31].

In the present study, despite no incidences of cirrhosis or extrahepatic obstruction of the portal vein, splenic enlargement was associated with higher serum IL-6. There are various potential causes of splenomegaly, including hematological, hepatic, infectious, congestive, and inflammatory conditions. The mechanism or mechanisms underlying splenic enlargement vary based on etiology [32]. It has been reported that splenomegaly is observed in 20% of cases involving AIP, probably due to splenic and portal vein involvement, and that steroid therapy can reduce the size of the spleen [33]. Cheng et al. described an AIP patient with splenomegaly in whom splenectomy led to the improvement of pancreatic swelling. Based on that case they speculated that the spleen may be involved in the pathogenesis of AIP because it is an immune organ. Additionally, IL-6 transgenic mice exhibit splenomegaly that is improved via the administration of anti-IL-6-receptor monoclonal antibody [34]-[35], suggesting that splenomegaly may be induced by increased IL-6. From the aspect of comparison between IgG4-RD and MCD, Sasaki et al. reported 48.5% of patients with MCD showed splenomegaly, while patient with IgG4-RD never exhibited splenomegaly [36]. In our study, 16% of patients who were diagnosed as IgG4-RD had splenomegaly, yet the mechanism involved in splenomegaly in IgG4-RD patients and the issue as to whether IgG4-RD accompanied with splenomegaly is “true” IgG4-RD remain unclear, and further study is necessary.

There were a few limitations in the current study. Firstly, the study was retrospectively conducted. Secondly, limited data about serum IL-6 levels after steroid therapy were available. Thirdly, due to small sample size, it was impossible to perform multivariate analysis for enhancement of the evidence for a link between elevated IL-6 levels and involvement of organs, such as the bile duct or liver. To resolve these limitations, a prospective well-designed multicenter study is needed.

In conclusion, serum IL-6 level at the onset of IgG4-RD may be significantly correlated with clinical inflammatory parameters such as serum levels of CRP, hemoglobin, and albumin, and it may also be associated with involvement of the bile duct and liver. With regard to serum IL-6 levels, IgG4-RD diagnosed in accordance with the current diagnostic criteria may include heterogeneous groups characterized by different underlying pathogenic components. Further studies in IgG4-RD patients with elevated IL-6 levels are needed to elucidate the pathogenesis of IgG4-RD and to develop accurate diagnostic criteria enabling the identification of “true” IgG4-RD.
